# Formaldehyde1Read before the Illinois State Veterinary Medical Association, November 10, 1900.

**Published:** 1901-03

**Authors:** E. L. Quitman

**Affiliations:** Chicago, Ill.


### FORMALDEHYDE.[1]

By E. L. Quitman, M.D.C.,
CHICAGO, ILL.

Synonyms : Formalin, formal. These terms are applied to a 40 per cent. solution of formic aldehyde in water. It is a gaseous product, made by subjecting methyl alcohol (wood alcohol) to oxidation. It is readily absorbed by water, at least to a 40 per cent. solution.

It should only be used externally, for when used internally it produces a variety of toxologic conditions, notably inanition, bronchopneumonia, a catarrhal condition of the bowels, and sometimes acute nephritis. Externally, I would typify it as the most powerful antiseptic, disinfectant, and deodorizer we have. It is also a powerful constrictor of arterioles and capillaries, frequently allowing of practically a bloodless operation of parts where there are no large bloodvessels. So powerful is it that a 1 : 20,000 solution is said to destroy the various pathological germs if sufficient exposure is allowed.

As a general antiseptic for surgery and in the treatment of wounds I recommend its use in a solution of 0.25 to 0.50 per cent. For very foul wounds or where sepsis is great from 0.50 to 1 per cent. As a destroyer of small growths, such as grapes, from mud fever or greasy heel, fistulous tracts, as seen in quittors, poll-evil, etc., from 2 to 10 per cent. When using it in this last strength I would admonish you to be careful and apply it to the diseased area only. The handiest method by which to apply it to growths of various kinds is to wrap a piece of absorbent cotton around a probe, or "ball" up a piece and catch it in an artery forceps, dip it into the solution, pressing off the surplus and swab the parts.

In fistulous openings it may be injected, using care not to have it flow over the surrounding parts or having them protected by a heavy coating of vaseline. If used too strong it causes dry gangrene. The gangrenous portion will remain in place for months unless forcibly removed, there seeming no tendency to slough.

Of this property to cause dry gangrene I often make use of in treating quittors and similar conditions. I enlarge the openings and inject a 2 to 10 per cent. solution for several days until dry gangrene of the part is established, and then curetting or removing

[1] Read before the Illinois State Veterinary Medical Association, November 10, 1900.

the areas, which may be whittled off like dead bark or wood, without pain.

Wounds healed under its use leave but very little if any scar, where ordinarily a larger one would remain, inasmuch as it possesses wonderful properties in stimulating the growth of hair. To give you an idea as to its power in this regard, I will state one case where a horse broke through a floor tearing the skin and subcutaneous tissues to such an extent as to entirely denude the front leg from the knee to the fetlock, exposing the bone and sheaths of tendons. Now, ordinarily this would have simply cicatrized over, leaving the leg practically bare of hair, as the skin could not be sewed up, and was removed. This case was dressed once daily with a 0.75 per cent. solution of formaldehyde (Merck) in water, applied on absorbent cotton, and while it took about two months to heal on account of the horse chafing it at every opportunity, it left only a scar about two inches long and three-quarters of an inch wide.

In Chicago septic conditions of the feet running a very rapid course, resulting from nail, pricks, calkings, suppurating corns, etc., are very prevalent, and I am sure that since using this agent I have saved the lives of a number of horses that I would otherwise have lost, and where death would not occur by virtue of its rapid and profound action, it prevents the extensive necrosis that frequently follows in these conditions.

I use it in foot baths for nail-pricks, suppurating corns, etc., an ounce to each bucketful of water; if a poultice is used I put about an ounce in a poultice large enough to cover an entire hoof or apply a 1 per cent. solution on cotton to the parts, possibly injecting a 0.25 or 0.50 per cent. solution. I use it for my hands and instruments in all operations. To disinfect stables, etc., it may be sprayed into the air from an atomizer, or sheets or cloth may be saturated with it and hung around the premises, or it can be sprinkled around from an ordinary sprinkling can or by means of a broom. For this about a 20 per cent. solution is ordinarily used, and the premises should be sealed up for six to ten hours. The fumes are somewhat irritating to the eyes and air passages, but do not cause any harm.

For hardening and preserving anatomical specimens place them for twenty-four to forty-eight hours in a 2 to 4 per cent. solution, then into a 10 per cent. solution.

It never causes any danger by absorption, its only trouble being that if used too strong it causes dry gangrene.

Try it, and I am sure it will become your favorite antiseptic.

